# Application of Ultrasonography in Neurogenic Dysphagia: A Systematic Review

**DOI:** 10.1007/s00455-022-10459-9

**Published:** 2022-05-13

**Authors:** Paola Potente, Alex Buoite Stella, Monica Vidotto, Michelle Passerini, Giovanni Furlanis, Marcello Naccarato, Paolo Manganotti

**Affiliations:** 1 Clinical Unit of Neurology, Department of Medicine, Surgery and Health Sciences, University Hospital of Trieste ASUGI, University of Trieste, Strada di Fiume 447, 34100 Trieste, Italy; 2 School of Speech and Language Therapy, Department of Medicine, Surgery and Health Sciences, University of Trieste, Trieste, Italy

**Keywords:** Dysphagia, Ultrasound, Muscle thickness, Hyoid displacement, Neurological diseases, Deglutition, Deglutition disorders

## Abstract

Swallowing disorders are common in neurological diseases, with dysphagia representing one of the most prevalent complications that may cause poor quality of life, reduce independence, and increase mortality. Rapid identification of dysphagia is necessary to reduce the risk of penetration and aspiration, and to early start rehabilitation protocols. Among the methods that can be used to evaluate dysphagia and its components, ultrasound imaging has been suggested to support the evaluation of dysphagia by providing measures of both static and dynamic anatomical components. The aim of this systematic review is to evaluate the usefulness of ultrasonography in neurogenic dysphagia according to current literature. From 2000 to 2020, 633 studies with the appropriate search terms for ultrasound and dysphagia were identified. After screening them, 10 studies were included in the qualitative analysis. Patients with the following neurologic conditions were studied with ultrasonography for dysphagia: Parkinson’s disease, muscle dystrophy, amyotrophic lateral sclerosis, and stroke. The main outcomes of ultrasonography were swallowing muscles thickness (e.g., tongue), and dynamic measures such as hyoid displacement. The different protocols used in the studies, as well as their outcomes, did not allow to provide standard procedures and normative or cut-off values in the presented diseases. Because there are a variety of tools, methods, and techniques that have been used in the studies that were reviewed, it is difficult to evaluate them using established standards. However, ultrasonography correlates well with clinical evaluation of dysphagia and therefore has prognostic and rehabilitation potential. Future studies should aim to develop and utilize a common interdisciplinary protocol that includes standard procedures and outcomes to define normative values applicable in the different conditions.

## Introduction

Swallowing is a physiological function that requires the coordination of several muscles located within the oral cavity, pharynx, larynx, and esophagus. It is the result of a complex sensorimotor behavior coordinated by a neural interplay at both cortical and subcortical levels [1–3]. The disturbance of the intake or transport of food from the mouth to the stomach is a symptom defined as dysphagia. This swallowing dysfunction is commonly linked to poor outcomes, including a higher risk of longer hospital stay, pneumonia, disability, and death [4, 5].

Swallowing can be described as the result of complex interactions between sensory inputs and motor outputs, involving both voluntary and involuntary processes. Sensory inputs from peripheral structures are processed in sensorimotor cortical areas and brainstem structures, modulating the motor integration of the swallowing network [6]. Swallowing is composed of three major phases: oral, pharyngeal, and esophageal. The first phase is voluntary and involves lips, teeth, muscles of mastication, and tongue, while the second and third phases are involuntary. In particular, the pharyngeal phase is characterized by a reflex response, and it represents the most complex phase of swallowing since it requires the coordination of pharyngeal and laryngeal muscles, as well as tongue and suprahyoid muscles [1, 3, 7].

In neurological diseases, dysphagia is often a consequence of central nervous system, peripheral nervous system, neuromuscular junctions, and muscles impairment. In Stroke, dysphagia may be present in 51 to 78% of patients [4, 8], while in Parkinson’s disease (PD), oropharyngeal dysphagia occurs in 80% and 95% of patients in the early and advanced stage of the disease, respectively [9]. Dysphagia also occurs in many other neurological diseases such as amyotrophic lateral sclerosis (ALS), with a prevalence of 80% in late stages [10], multiple sclerosis (MS) in 31.3% of overall patients, Duchenne muscular dystrophy (DMD) with a prevalence of 96.8% [11], and dementia with a prevalence varying between 13 and 57% [12].

Swallowing impairments can be assessed by clinical or instrumental testing. Clinical evaluation is usually conducted by the Speech and Language Pathologist (SPL) through the bedside swallowing examination (BsSE) [13]. Instrumental testing most commonly includes the videofluoroscopic swallowing study (VFSS) and the fiberoptic endoscopic evaluation of swallowing (FEES). Both tests are well validated. However, exposure to radiation, patients collaboration and invasiveness may represent a limitation to its wide application in the clinical setting [14].

In the last few years, some studies have applied ultrasonography (US) to evaluate swallowing structures, finding a good correlation with swallowing function [15–20]. US has several advantages: it is non-invasive, widely applied in clinical practice because of its low cost, portability, and it is radiation free. It may also enable to use of regular food, too [15–18]. Relevant literature describes this promising tool to evaluate morphological and biomechanical (dynamic) systems involved in swallowing, focusing on tongue, suprahyoid muscles, larynx, and hyoid bone analysis [15–21].

The tongue is a fundamental skeletal muscle for mastication, manipulation, containment, and transport of bolus to the tongue base during the oral phase of swallowing. Oro-pharyngeal dysphagia is a common sequela of neurological diseases related to a reduction of strength and endurance of the tongue and resulting in lateral sulcus residue, poor bolus formation, and mastication during the oral phase, as well as vallecular residue and aspiration during the pharyngeal phase [22]. Ultrasonography research applied to tongue studies uses real-time B-mode or combined B + M-mode images with a submentally placed sector transducer (3–7 MHz) to obtain temporal and spatial information. Quantitative information can be acquired with temporal or spatial measure and by measuring tongue shape changes during swallowing or tongue thickness in a static or dynamic position, respectively [19]. Moreover, qualitative data can be obtained by recording the difference in tissue echo intensities, since lingual myoarchitecture variations can detect regional tissue changes caused by pathologies [23]. Quantitative and qualitative studies of suprahyoid muscles are also included in several ultrasonography studies as a contraction of these muscles causes excursion of the hyolaryngeal complex during swallowing [15]. Hyoid bone motion is an important, mobile biomechanical marker of swallowing. Its motion is important for the control of tongue movement, the opening of the upper esophageal sphincter, and the tilting of the epiglottis to close and protect the airway from entry of foreign material [24]. Hyoid imaging typically requires a submentally placed sector transducer (3.5–5.0 MHz). The hyoid can be clearly displayed using a midsagittal B-mode ultrasound as a dark band of non-echoic acoustic shadow. Its movement and temporal coordination provide clinical information since differences in hyoid bone displacement are associated with an increased risk of penetration and aspiration of liquid and food [25, 26].

However, due to the lack of a common consensus and specific guidelines on US use to assess swallowing impairments, such studies have reported different protocols and outcomes, and some of them used heterogeneous clinical populations, making it difficult to reproduce, generalize, and compare those data [27–29].

As such, the aim of this systematic review is to evaluate the usefulness of ultrasonography in neurogenic dysphagia according to current literature.

## Methods

**Fig. 1:**
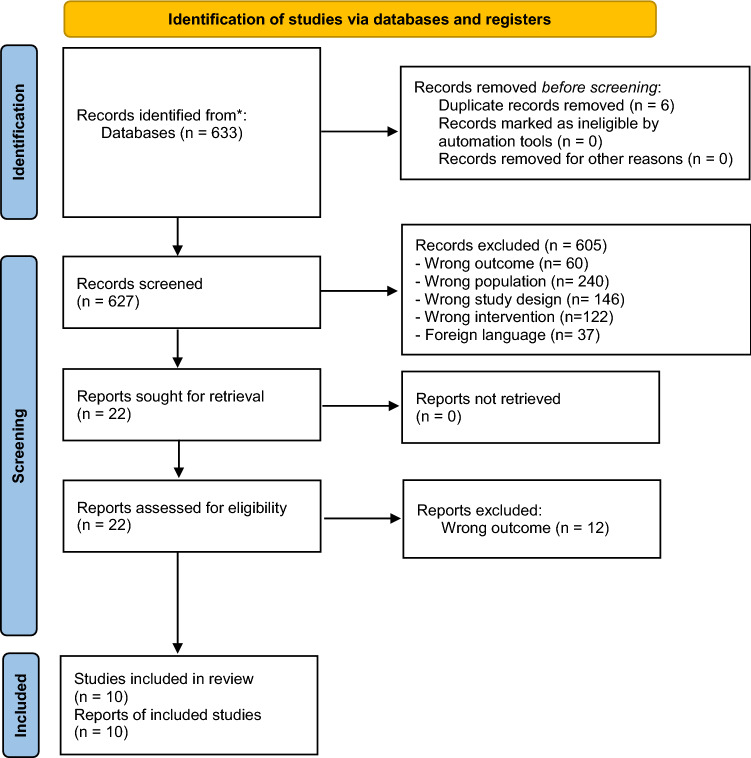
Flowchart of the study selection process. PRISMA 2020 Flow Diagram for search of literature and assessment of eligibility for studies included in the review. A total of 633 records were identified and screened, among which 10 papers were included in the qualitative synthesis and presented from [55] For more information, visit: http://www.prisma-statement.org/

The Cochrane Handbook and PRISMA guidelines (2020 updated version) were followed for the methodology and reporting of the present review [30, 31]. A complete flowchart of the study studies selection process [32] is shown in Fig. 1.

### Information Sources, Search, and Study Selection

Three different databases (Medline, Web of Science, and Embase) were investigated from inception to 01.11.2020. The search strategy was structured according to the PICO (Population Intervention Comparison Outcome) method as follows: P (“Dysphagia”) AND I (“Ultrasound”) AND O (“Muscle” OR “Hyoid”).

Two independent co-authors (PP and MV) checked the eligibility of studies. The web application Rayyan (Qatar Computing Research Institute, Doha, Qatar) was used to explore and filter results independently, identifying records in a blinded standardized manner. Full texts of studies whose title and abstract met inclusion criteria were obtained and screened. If the two authors disagreed on the inclusion of study, a third author (ABS) was asked to arbitrate.

### Eligibility Criteria

We defined specific exclusion criteria for the study selection process, namely,

1. Studies not written in English (labeled “wrong language” *n* = 37), or study data reported in multiple publications (*n* = 6)
2. Reviews, meta-analysis, study protocols, case reports, letters to the editor, errata, and conference proceedings (labeled “study design” *n* = 146)
3. Studies on non-adults with neurological diseases, in vitro or on animal models (labeled “wrong population” *n* = 239)
4. Ultrasound not performed, measures not reported, or other anatomical structures not involved in swallowing (labeled “wrong intervention” *n* = 122 and “wrong outcome” *n* = 64)

### Data Collection and Analysis

A pilot test was conducted on three different randomly selected studies, asking two co-authors to independently extract data (PP, MV). A third author (ABS) assessed the accuracy of data extraction. From each included study, data were extracted including (i) patients’ characteristics and demographics, (ii) disease and dysphagia classification, and (iii) ultrasound measure. Careful assessment of ultrasound measures was considered for homogenous protocols and to insure a clear methodological approach. All data were clearly reported in the manuscript.

## Results

### Study Selection, Characteristics of the Sample, and Testing Protocol

The total search yielded 633 records, including 6 duplicate records. The remaining 627 records were assessed for eligibility by carefully checking titles and abstracts, and 605 records were excluded according to the eligibility criteria described above in the Methods section. Full texts were obtained and screened for 22 articles, among which 12 were further excluded mainly due to “wrong outcome.” When subgroups were present within a study, they were clearly reported in this systematic review and data were considered separately. In total, 292 patients from 10 studies with a diagnosis of neurological diseases were included. Among this sample, 181 were diagnosed with stroke (both in the acute and chronic phase), 47 with Parkinson’s disease, 46 with muscle dystrophies, and with 18 amyotrophic lateral sclerosis. Ultrasound measurements were performed to assess tongue, facial, and suprahyoid muscle thickness and echogenicity, and to assess hyoid displacement features.

### Qualitative Synthesis of Ultrasound Evaluation of Swallowing in Different Neurological Diseases

#### Stroke

Ultrasound assessment in the stroke population was used to evaluate functional properties of oro-laryngeal structures in dysphagia. Ming-Yen Hsiao et al. [33] conducted an ultrasound examination, using a 3.5 MHz convex array, on 90 participants: 60 stroke patients and 30 healthy participants. Stroke patients were divided into two groups according to the functional oral intake scale (FOIS). Group1 included 30 patients graded as dependent on tube feeding (FOIS 1–3), while group2 included the other 30 patients graded as oral intake (FOIS 4–7). Tongue thickness was measured at the central plane bisecting the angle formed by the acoustic shadows of the hyoid and mandible. Then, the mean change in tongue thickness was calculated by tracing the maximum and minimum tongue thickness change during swallowing of 5 mL of water. The measure did not differ between the control group and the oral intake group, whereas a significant difference was observed among the tube-feeding depending group, the oral intake group, and the control group (group1 vs. group2 *p* = 0.002; group1 vs. control *p* =  < 0.001). Furthermore, using the mandible as a reference point, the mean hyoid bone displacement was significantly different among the tube-feeding depending group, the oral intake group and the control group (group1 vs. group2 *p* = 0.001; group1 vs. control *p* =  < 0.001). This outcome highlights an inadequate larynx elevation, increasing the risk of aspiration and that reflects a severe impairment of swallowing function. Most of the studies included in this review investigated the hyoid-larynx approximation and laryngeal elevation as they are directly associated with bolus passage through the pharyngeal esophageal segment, closure of cricopharyngeal muscle and airway protection [34]. Huang et al. [35] explored the ultrasound examination of hyoid-larynx approximation and its application on 55 participants. 40 stroke patients, 20 with dysphagia and 20 without, were matched with 15 healthy subjects. A 10 MHz curved linear array was used to measure the distance between the thyroid cartilage and hyoid bone, at rest before (resting distance) and during swallowing. Hyoid-larynx approximation was obtained by subtracting the shortest distance between these two structures during swallowing from the resting distance. Moreover, the change percentage of hyoid-larynx approximation, obtained by dividing the approximation distance by the resting distance, was calculated. The change percentage of hyoid-larynx approximation was significantly greater in normal subjects than in Stroke patients with normal swallowing (*p* = 0.028). The distance between the hyoid bone and thyroid cartilage during swallowing was significantly shorter in healthy subjects than stroke patients without dysphagia and in Stroke patients with dysphagia, respectively, *p* = 0.006 and *p* = 0.008. No significant differences were observed in approximation distance, even though it was greater in normal subjects compared to both stroke groups. Hyoid-larynx approximation distance (cm) and degree (%) were also investigated by Picelli et al. [36] using a real-time B-mode ultrasound with a 6 MHz linear probe on 19 acute stroke patients, with and without dysphagia. Clinical bedside screening for dysphagia was assessed with the Gugging Swallow Screen (GUSS) and Functional Oral Intake Scale (FOIS). A significant direct association was found between FOIS and both hyoid-larynx approximation distance (*p* = 0.001) and degree (*p* = 0.005). Also, GUSS score showed a significant direct association with hyoid-larynx approximation distance (*p* = 0.008) and degree (*p* = 0.004). As to US evaluation, significant differences were found between dysphagic and not dysphagic patients in both hyoid-larynx approximation distance (*p* = 0.013) and degree (*p* = 0.011).

Lee et al. [37] analyzed the hyoid bone displacement using a 1–5 MHz curved probe as already done by previous authors. Yet, unlike them, Lee et al. estimated the difference between the mandible and the hyoid bone at rest and during swallowing to obtain the hyoid bone displacement and percentage of its displacement (delta value). As many as 52 subjects with dysphagia, 45 stroke and 7 with other conditions, were included. All of them underwent FEES and were divided in three groups according to the penetration–aspiration scale (PAS): non-aspirators (*n* = 21), penetrators (*n* = 20) and aspirators (*n* = 11).

Moreover, since post-swallowing pharyngeal residue is an important risk factor for the occurrence of aspiration [38], the researchers considered a second split based on the amount of residues in the pyriform sinus and vallecular fossa after swallowing: Grade 0 as no residue, grade 1 is < 10% of residue, and grade 2–3 are > 10% of residue.

According to the penetration scale, the difference in the mean value of hyoid bone displacement and delta value among groups was statistically significant (*p* < 0.001). Measures were significantly (*p* < 0.001) shorter and smaller (*p* = 0.001) in both the penetrators and aspirators groups compared to the non-aspirators group, while the displacement distance and delta value in the aspirators group were significantly (*p* = 0.001) shorter and (*p* = 0.002) smaller than those in the penetrators group. According to the residues in pyriform sinus and vallecular fossa, hyoid bone displacement was found to be lesser in the group with a larger amount of residue. All comparisons done between these groups using a post hoc test showed statistically significant results (*p* = 0.036 and *p* < 0.001, respectively). Thus, according to the authors [37], this means that US could be a predictable tool for post-swallowing pharyngeal residue level. Ultrasound was also applied to investigate the dynamics of swallowing. Söder and Miller [39] used a linear transducer on 20 stroke patients and 20 healthy subjects to evaluate the intrasubject variability in the entire duration (ED) of tongue movement during swallowing and duration of tongue movement during the oral transport stage (OTS) in the patients and control groups. The patients group revealed a shorter mean ED and OTS than the control group but these did not differ significantly, as did not the mean OTS. However, the patients group presented a significantly wider range (*p* = 0.0189) compared to the control group, and a significantly greater standard deviation during the OTS (*p* = 0.024). Findings from this population are summarized in Table 1.

**Table 1:** Stroke

Reference	Object of evaluation	Control group measure	N	Patients group measure	N
				Stroke patients	
Söder and Miller [39]	Mean of ED (ms)	2133.2	10	1990.31	10
	Mean of OTS (ms)	912.09		1031.12	
	Standard deviation of the duration of ED	358.3		388.45	
	Standard deviation of OTS	230.1		397.67	
	Range of duration of ED	1264		1353	
	Range of duration of OTS	824.6		1361	

				Stroke		
				No dysphagia (*n* = 20)	dysphagia (*n* = 20)	
Huang et al. [35]	Resting distance hyoid-thyroid (cm)	3.18 ± 0.35	15	3.3 ± 0.4	3.43 ± 0.59	40
	Shortest distance hyoid-thyroid (cm)	1.67 ± 0.18		1.87 ± 0.21	2.26 ± 0.59	
	Approximation distance hyoid-larynx (cm)	1.51 ± 0.28		1.43 ± 0.43	1.16 ± 0.42	
	Hyoid-larynx approximation degree (%)	47.2 ± 4.9		42.6 ± 8.3	34.0 ± 10.9	

				FOIS 4–7 (*n* = 30)	FOIS 1–3 (*n* = 30)	
Hsiao et al. [33]	Tongue thickness change (cm)	1.1	30	1.0	0.9	60
	Hyoid bone displacement (cm)	1.7		1.6	1.3	

				Stroke (*n* = 45) + Others condition (*n* = 7)		STROKE (*n* = 45) + Others condition (*n* = 7)		
				Non-aspirators (*n* = 21)	Penetrators (*n* = 20)		Aspirators (*n* = 11)	
Lee et al. [37]	Resting distance (cm)	**–**		3.93 ± 0.54	3.78 ± 0.24		3.72 ± 0.40	52
	Shortest distance (cm)	**–**		2.44 ± 0.49	2.62 ± 0.29		2.94 ± 0.41	
	Hyoid bone displacement (cm)	**–**		1.59 ± 0.27	1.15 ± 0.28		0.8 ± 0.1	
	Delta value ^a)^ (%)	**–**		40.2 ± 8.5	30.4 ± 7.1		21.8 ± 4	

				Pyriform sinus residue			
				Grade 0 (*n* = 15)	Grade 1 (*n* = 25)	Grade 2–3 (*n* = 12)	
	Resting distance (cm)	**–**		3.90 ± 0.42	3.77 ± 0.30	3.89 ± 0.48	
	Shortest distance (cm)	**–**		2.51 ± 0.55	2.53 ± 0.29	2.95 ± 0.43	
	Hyoid bone displacement (cm)	**–**		1.48 ± 0.36	1.25 ± 0.32	0.94 ± 0.38	
	Delta value ^a)^ (%)	**–**		38.4 ± 11.2	33.1 ± 7.5	24.0 ± 8.0	

				Vallecular fossa residue			
				Grade 0 (*n* = 11**)**	Grade 1 (*n* = 24)	Grade 2–3 (*n* = 17)	
	Resting distance (cm)	**–**		3.96 ± 0.43	3.83 ± 0.37	3.76 ± 0.36	
	Shortest distance (cm)	**–**		2.50 ± 0.60	2.56 ± 0.39	2.79 ± 0.37	
	Hyoid bone displacement (cm)	**–**		1.59 ± 0.35	1.29 ± 0.35	0.97 ± 0.27	
	Delta value ^a^ (%)	**–**		40.8 ± 11.8	33.5 ± 8.3	25.8 ± 6.6	

				No dysphagia	dysphagia	
Picelli et al. [36]	Hyoid-larynx approximation distance (cm)	**–**		1.4 ± 0.4	1.1 ± 0.1	19
	Hyoid-larynx approximation degree (%)	**–**		47.3 ± 12.1	34.9 ± 5.0	

^a^Hyoid bone displacement/ Resting distance × 100

*ED* Entire swallow duration, *OTS* Oral transport stage, *FOIS *Functional Oral Intake Scale

In summary, the dynamics of the hyoid-laryngeal system were studied in the acute and subacute stroke population in the US evaluation of swallowing function, too. Post-stroke dysphagia (PSD) represents a common complication affecting a high number of patients in the first few hours and days after a stroke [40, 41]. In the acute phase, usually dysphagia is screened in first instance and then clinically evaluated by the speech language pathologist. US measure of hyoid-larynx approximation distance and degree showed a significant direct association with clinical tools as FOIS and GUSS as defined by Picelli et al. [36]. This highlights how US could be a helpful tool to a better and objective examination of swallowing for the bedside screening in the acute phase. Hyoid-larynx approximation was also studied in the chronic phase. In the acute phase as well as in the chronic phase of stroke. Reduction in hyolaryngeal approximation was found to have good sensitivity and specificity for the clinical detection of dysphagia. Moreover, changes in tongue thickness and hyoid bone displacement during swallowing were reported in severely dysphagic, tube-feeding-dependent stroke patients [33]. US is an additional method that can be used to predict the need for tube feeding in severe dysphagia.

#### Parkinson’s Disease (PD)

Ultrasound evaluation has been reported in two studies. Anatomical characteristics were evaluated in 24 PD patients who reported dysphagic characteristics, later confirmed with VFSS, and compared to 24 matched healthy controls by Oh et al. [42]. In this study, ultrasound assessment was conducted using a 2–6 MHz curved-array transducer. The participants were examined in a comfortable upright sitting position with a neutral head position. The measures were taken in a midsagittal plane, recording a longitudinal view from the tongue base to the thyroid cartilage. Tongue thickness was found to be smaller in PD patients compared to controls; however, the difference did not reach statistical significance (*p* = 0.292). Moreover, hyoid-thyroid approximation, which identifies the laryngeal elevation following the hyoid bone movement (usually lower in patients with dysphagia), was measured. The time to hyoid-thyroid approximation, which represents the interval between the initiation of tongue movement and the shortest distance of hyoid-thyroid approximation, was also measured as a marker of oropharyngeal swallowing dysfunction. These two measurements were both greater in PD patients compared to controls, although only the latter reached statistical significance (*p* = 0.149 and *p* = 0.048, respectively). In the second study, conducted by Grunho et. Al [43], the oropharyngeal phase duration of water (wet swallows) and saliva only (dry swallows) was investigated in 23 PD patients with corticobasal syndrome and compared to 28 matched healthy controls. A 5 MHz ultrasound transducer was placed submentally in the midsagittal plane to visualize the movements of the tongue, the floor of the mouth muscles, and hyoid bone in the subjects. No significant differences were found in the dry swallow duration (*p* = 0.200), while wet swallow was significantly prolonged compared to the healthy controls (*p* = 0.001). According to the author, the prolonged duration of the phase could be partially explained by apraxia, since dry swallow requires more voluntary control and effort than wet swallows. Findings from this population are summarized in Table 2.

**Table 2:** Parkinson’s disease

Reference	Object of evaluation	Control group measure	N	Patients group measure	N
Grunho et.al [43]	Duration of oropharyngeal phase of wet swallow (s)	2.64 ± 1.7	28	2.73 ± 1.0	23
	Duration of oropharyngeal phase of dry swallow (s)	3.44 ± 2.2		6.77 ± 4.5	
Oh et al. [42]	Tongue thickness(cm)	4.42 ± 0.46	24	4.27 ± 0.51	24
	Hyoid-thyroid approximation (cm)	1.19 ± 0.34		1.37 ± 0.5	
	Time to hyoid-thyroid approximation (ms)	1.53 ± 0.87		2.4 ± 1.4	

In summary, according to the studies conducted on Parkinson’s disease patients, ultrasonography proved to be a useful tool to detect oropharyngeal dysfunction through the evaluation of time to hyoid-thyroid approximation.

#### Muscular Dystrophy

Two studies evaluated the echogenicity and thickness of several muscles involved in swallowing. Engel-Hoek et al. [44] divided 24 patients into three groups according to the clinical stages of DMD: early and late ambulatory stage (AS), early non-ambulatory stage (ENAS), and late non-ambulatory stage (LNAS). For submental muscles assessment, an 8.5 MHz linear array was placed in transverse position. The echo intensity of the digastric, geniohyoid, superior, longitudinal, and transversus tongue’s muscles showed a gradual increase. Specifically, a significant difference among the three DMD stages was found for the geniohyoid and tongue muscles. Moreover, in ENAS and LNAS, the tongue thickness was abnormal in 70% of the patients, showing a significant correlation between tongue thickness and echo intensity of superior longitudinal tongue muscle (*p* = 0.001). The increased echo intensity (*z* score > 2) in LNAS of the digastric and tongue muscles reflects degeneration of these oral muscles and explained the need for multiple swallows to clear the oral cavity when swallowing solid food, as evidenced in VFSS. Additionally, in this study, VFSS confirmed pharyngeal post-swallow residue starting from the AS and increasing in the ENAS and LNAS. In the second study, anatomical characteristics were assessed in 22 patients [45]. Echogenicity and thickness of orofacial muscles were compared between Duchenne muscular dystrophy and Becker muscular dystrophy in the early ambulatory stages. The array’s characteristics are not reported in the paper. Only the thickness of temporalis muscle was significantly greater in patients with BMD. This may suggest that BMD patients show early mastication and swallowing problems that require early clinical assessment of feeding and swallowing functions. Findings from this population are summarized in Table 3.

**Table 3:** Muscular dystrophy

Reference	Object of evaluation		Patients group measures		
			Duchenne muscular dystrophy		
			AS (*n* = 6) Mean (95% CI)	ENAS (*n* = 7) Mean (95% CI)	LNAS (*n* = 11) Mean (95% CI)
Lenie van den Engel-Hoek et al. [44]	tongue	MT	1.1 (0.6–1.5)	2.8 (1.0–4.6)	4.2 (2.0–6.5)
	m. digastricus left	MT	0.7 (0.1–1.3)	1.4 (0.5–2.3)	0.3 (− 0.9–1.4)
	m. digastricus right	MT	1.3 (− 0.4–2.7)	2.5 (1.4–3.5)	0.9 (0.7–2.4)
	m. digastricus left	EG	0.3 (− 1.1–1.7)	1.2 (− 1.-3.7)	3.6 (1.3–5.9)
	m. digastricus right	EG	1.3 (− 1.2–1.8)	1.4 (0.7–3.5)	3.3 (0.7–5.8)
	m. geniohyoid	EG	2.4 (− 0.8–5.5)	5.6 (0.8–10.3)	8.8 (5.3–12.3)
	m. superior longitudinal	EG	− 0.3 (− 0.8–0.8)	0.7 (-− 0.2–1.5)	3.7 (1.8–5.6)
	m. trasversus	EG	− 0.1 (− 1.9–1.7)	− 0.2 (− 1.2–0.8)	1.6 (0.7–2.5)

			Duchenne muscular dystrophy (*n* = 11) Mean ± SD	Becker muscular dystrophy (*n* = 11) Mean ± SD
Legarde et.al [45]	tongue	MT	− 0.2 ± 0.8	− 0.3 ± 1.0
	m. digastricus	MT	0.5 ± 1.2	− 0.1 ± 1.2
		EG	2.4 ± 2.2	2.1 ± 1.6
	m. geniohyoid	EG	0.8 ± 1.6	1.3 ± 1.3
	m. superior longitudinal	EG	0.9 ± 0.8	0.9 ± 0.9
	m. transverse	EG	2.2 ± 1.4	1.6 ± 1.2
	m. masseter	MT	− 0.0 ± 0.6	− 0.1 ± 0.6
		EG	3.5 ± 1.4	3.1 ± 1.3
	m. temporalis	MT	0.0 ± 1.0	1.0 ± 1.1
		EG	2.4 ± 1.8	2.3 ± 1.4

*AS* Early and late ambulatory stage, *ENAS* Early non-ambulatory stage, *LNAS* Late non-ambulatory stage, *EG* Echogenicity, *MT* Muscle thickness

In summary, Duchenne muscular dystrophy (DMD) and Becker muscular dystrophy (BMD) are neuromuscular disorders characterized by a progressive loss of muscle cells replaced by interstitial fat and fibrosis tissue. These muscle cells replacement caused orofacial muscle weakness, with the pseudohypertrophy of the tongue representing the main cause of mastication and swallowing impairment [46, 47]. According to the literature, the most useful ultrasonography measures in this population accounting for swallowing dysfunction are tongue thickness and echo intensity of suprahyoid muscles, especially digastricus and geniohyoid. Moreover, a gradual increase of tongue echo intensity showed a good correlation with a reduction in the duration of the oral preparatory and oral phases when solid foods are prepared. Moreover, the US findings demonstrated an early start of dystrophic changes in submental muscles and tongue in DMD, which became severely affected in later stages. Early detection of chewing problems through a timely evaluation and repeated US follow-up of swallowing structures could be fundamental to prevent choking and nutritional deficiency and aid clinicians to identify interventions.

#### Amyotrophic Lateral Sclerosis (ALS)

Nakamori et al. [48] investigated the validity of tongue ultrasonography for evaluating dysphagia in comparison to videofluoroscopy (VF). They compared 18 ALS patients with 18 matched healthy controls. The ultrasound examination was performed using a 3.5 MHz convex array transducer according to Tamura et al. [49] method to measure tongue thickness and a 7.5 MHz linear array transducer to evaluate fasciculation. Tongue thickness (distance between the upper and lower surfaces of the lingual muscles) of ALS patients was significantly lower than that of healthy controls (*p* = 0.016), which may reflect tongue muscle atrophy. It was also significantly associated with BMI in ALS patients (*p* = 0.003). Besides, the clinical group showed a significant decrease in tongue thickness over the course of the disease (*p* = 0.002), this result is in line with the progression of weakness and atrophy of bulbar muscles. VF temporal analysis confirmed that swallowing problems resulting from an increased oral preparation and transit time were significantly associated with tongue thickness (*p* = 0.040). Lastly, ultrasound results proved to be a useful tool to diagnose tongue muscle fasciculation*.* Findings from this population are summarized in Table 4.

**Table 4:** Amyotrophic lateral sclerosis

Reference	Object of evaluation	Control group measure	N	Patients group measure	N
Nakamori et al. [48]	tongue thickness (cm)	4.48 ± 0.3	18	4.19 ± 0.4	18

In summary, a progressive weakness of the tongue causes oropharyngeal dysphagia [50]. Only one study used US to assess dysphagia and its result was relevant only to tongue thickness. However, the ultrasound measure of tongue thickness turned out to be useful to identify swallowing dysfunction caused by tongue atrophy. This outcome reflected the preparatory and oral dysfunction caused by muscle mass reduction. Moreover, ultrasound measure could be applied ambulatory to register tongue thickness changes over the course of the disease as the muscle changes were correlated to dysphagia worsening. US may represent a useful tool also for the detection of tongue fasciculations as a non-invasive and less painful method, compared to electromyography. It may have a supporting role in the diagnosing of ALS [51, 52]. Specifically, in ALS, early stage fasciculations are important indicators of acute denervation and development of dysphagia in those patients [48].

## Discussion

Specific protocols to evaluate the swallowing function depending on the neurological pathology do not currently exist. Based on the literature findings, the use of US to analyze orofacial muscle thickness and hyoid-larynx movement may help to quantify swallowing function and serve as a complementary method for the anatomical and dynamic evaluation of several phases of swallow in neurological diseases. Indeed, US is able to detect structural changes caused by dystrophy or denervation of muscles, detect involuntary movements such as fasciculations, and provide dynamic video images of tongue motion during swallowing attempts. Hence, US should be used when evaluating swallowing in neurological disorders [20]. Generally, US represents a fast, non-invasive, repetitive, and low-cost tool that provides objective measures of deglutition. One of the main advantages is the bedside application and the evaluation of swallowing using natural food and liquids, providing an ecological outcome. Nevertheless, US might also have some disadvantages that should be considered; for example, the transducer positioning and the lack of precise anatomical markers may modify the outcomes. Also, performing reliable measures requires specific and extensive training, in particular with patients with severe conditions (including cognitive alterations) [53]. In the included studies, transducers’ characteristics were often different, within the same clinical population, too. This may represent a limitation for the generalizability of the findings, since US does not only depend on the operator but also on the acquisition device and setting. Against this background, suggesting normative values from the included study that may help to define clinical alterations is not possible. Additionally, the studies were characterized by different outcomes in most cases, and due to the paucity and the relatively small sample sizes of studies, single findings should not be considered as cut-off values. As such, future studies should aim to propose standard and common protocols to test neurogenic dysphagia with US, and to suggest normative values to help identifying pathologically and at-risk conditions.

## Conclusions

Dysphagia has a high incidence in neurological diseases [12]. In the last few decades, ultrasonography has been applied to assess swallowing function in this field and studies mainly focused on tongue thickness and hyoid bone displacement [54]. Ultrasound is a helpful method to assess swallowing dysfunction during a clinical evaluation in several neurological diseases. This technique can be used to understand functioning of oropharyngeal anatomy and to visualize tongue muscle anatomy and suprahyoid muscle function. It can display hyolaryngeal displacement and duration of a swallow along with neuromuscular dysfunction.

Because there is a wide variety of ultrasound systems and transducers, the clinician needs to be trained to correctly utilize each system. Ultrasound is a low-cost, portable, non-invasive tool that can use any food material in a swallowing study. Speech language pathologists can be easily trained to use this technique for clinical evaluations and rehabilitation of neurologic dysphagia.
